# Supplementary material for: Astrocytes monitor cerebral perfusion and control systemic circulation to maintain brain blood flow
Source: Nat Commun. 2020 Jan 9;11:131. doi: 10.1038/s41467-019-13956-y (PMC6952443; doi:10.1038/s41467-019-13956-y)
Supplement: Supplementary file 1 — Supplementary Information [file 41467_2019_13956_MOESM1_ESM.pdf]

Supplementary Information

Astrocytes monitor cerebral perfusion and control systemic circulation to maintain brain blood flow

Marina et al.

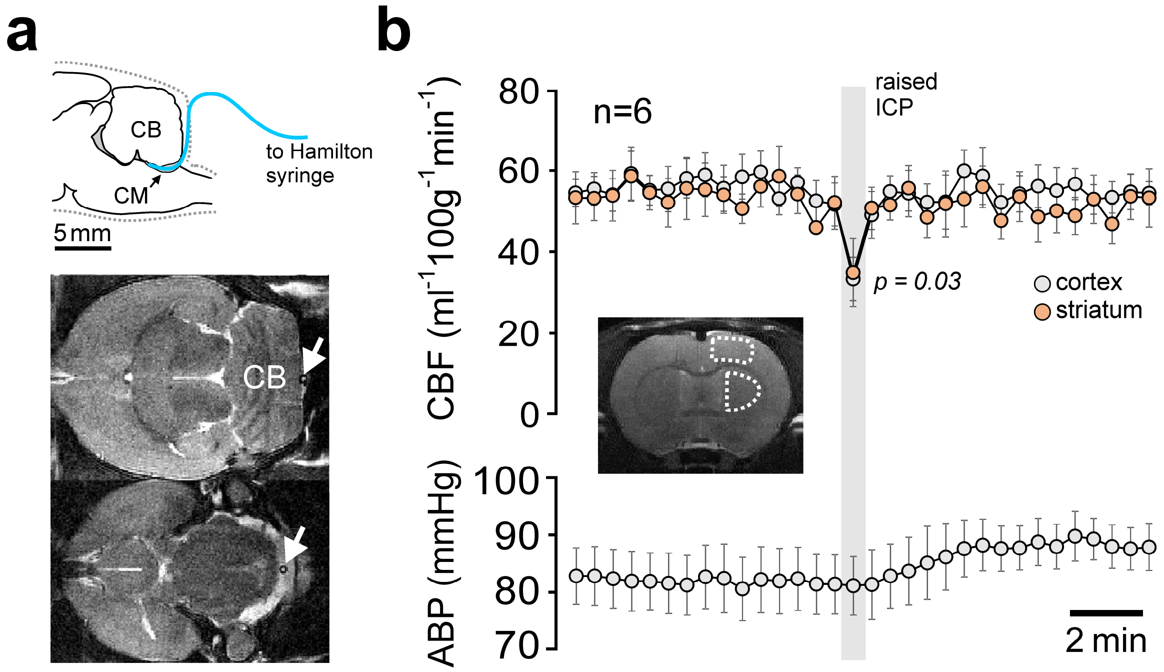

Supplementary Figure 1| Increase in intracranial pressure (ICP) acutely reduces the cerebral blood flow (CBF). (**a**) Due to the constrains of the MRI scanner environment, the rat brain ventricular system was accessed via a cannula implanted into the cisterna magna (CM) and connected using a saline-filled catheter to a Hamilton syringe for the delivery of the experimental stimulus. The experimental setup was pre-calibrated outside of the MRI scanner to deliver increases in ICP by 10-15 mmHg for 30-60 s. During imaging, the experimental stimulus of this duration was applied for the purpose of measuring acute CBF changes to ICP increases that occur prior to the development of the compensatory systemic cardiovascular response. High resolution magnetic resonance scans of the rat brain illustrate the positioning of the cannula (arrows). CB, cerebellum. (**b**) Summary data (means ± s.e.m.) illustrating the effect of raised ICP (by ~10 mmHg) on CBF in the cortex and the striatum. *Inset*: High resolution magnetic resonance scan of the rat brain with regions of interest outlined. ABP, arterial blood pressure.

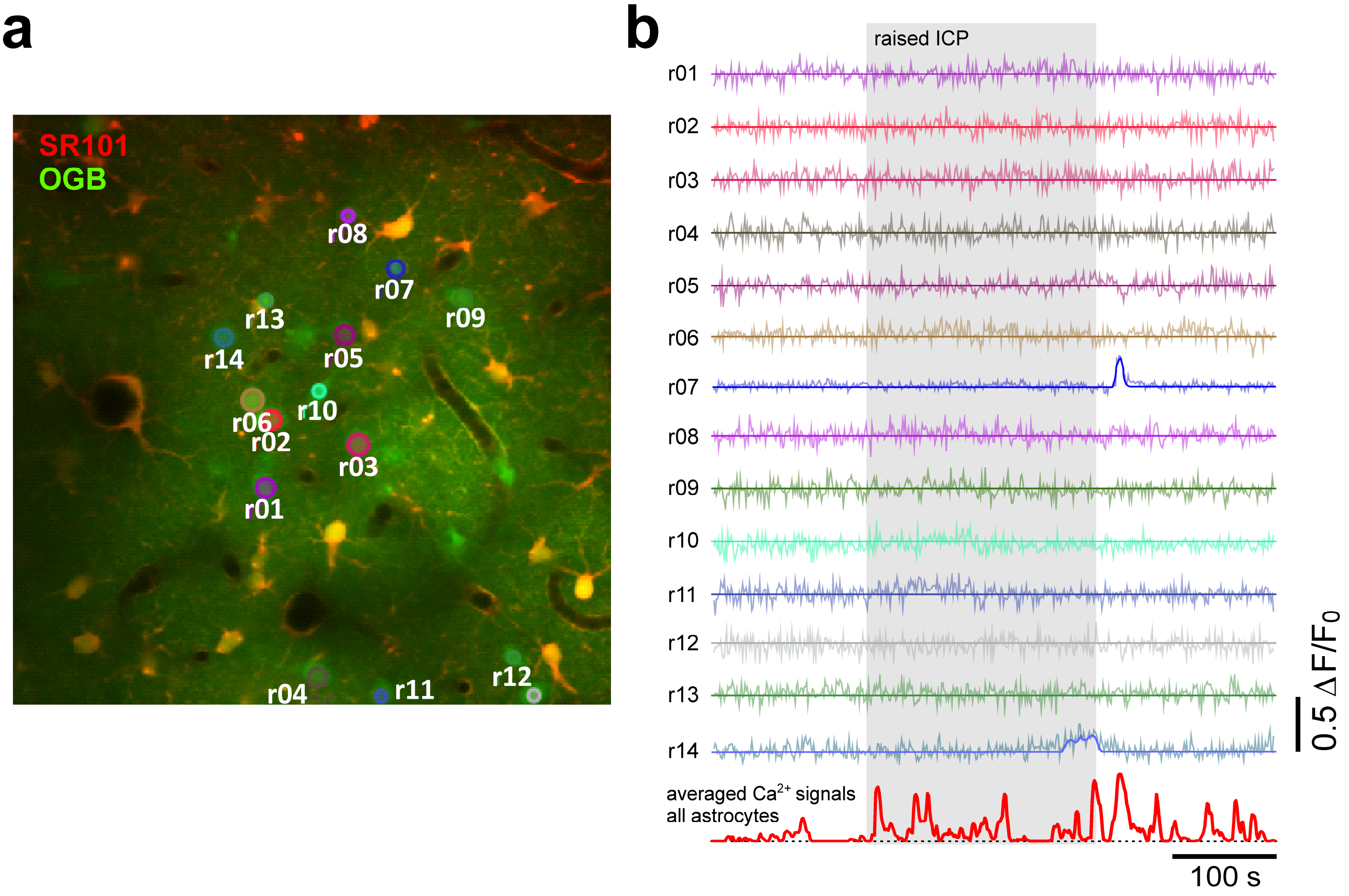

Supplementary Figure 2| (**a**) Identification of cortical cells that lack sulforhodamine 101 (SR101) labelling – putative neurons. Regions of interest (r1-r14) corresponding to neuronal cell bodies were detected by automated software. (**b**) Representative traces illustrate changes in Oregon Green BAPTA (OGB) fluorescence ($[Ca^{2+}]_i$) in the regions of interest identified as neurons before, during and after the period of raised ICP. Trace at the bottom illustrates an average of Ca^{2+} responses in all the astroglial compartments recorded in this experiment.

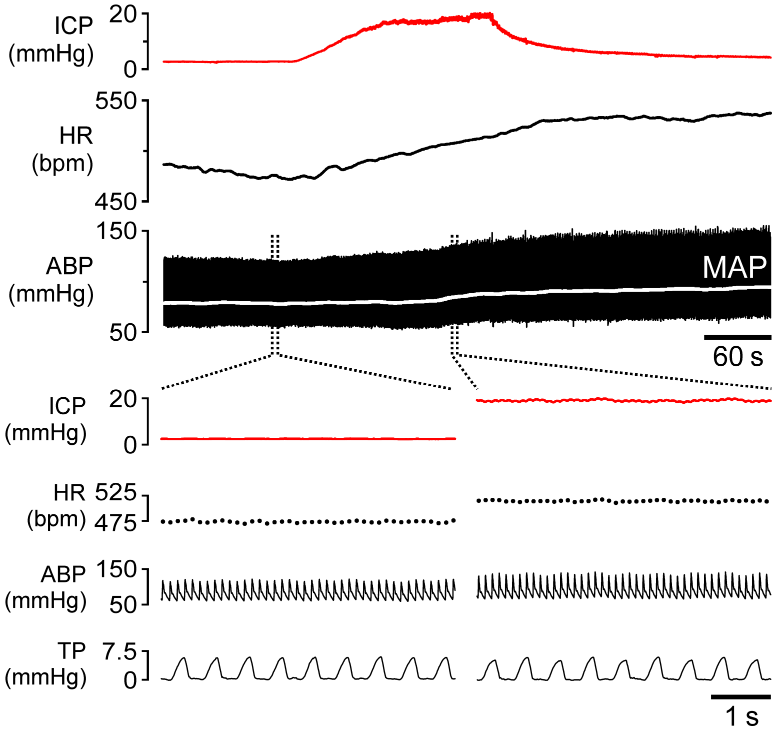

Supplementary Figure 3| Raw data illustrating changes in intracranial pressure (ICP), heart rate (HR), systemic arterial blood pressure (ABP) and tracheal pressure (TP; stability of TP indicates that there was no ventilatory response since the neuromuscular blockade was applied) induced by an increase in ICP and recorded simultaneously with the imaging data presented in Figure 2a-c.

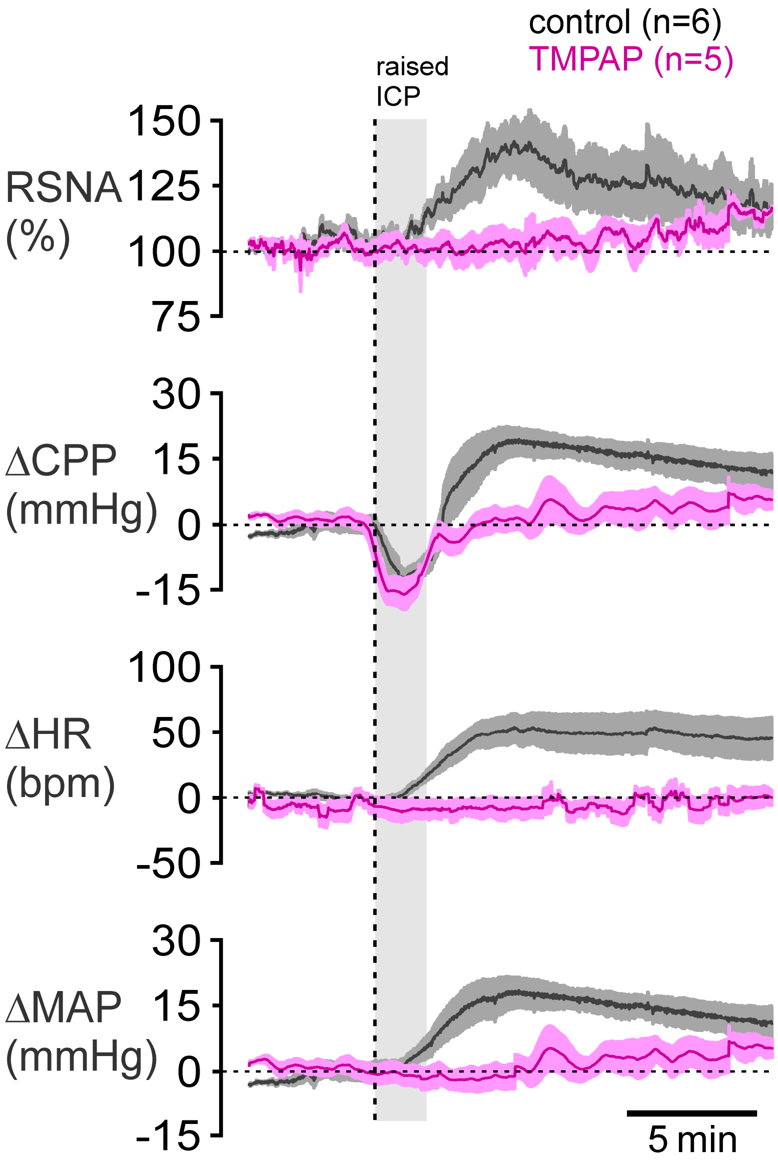

Supplementary Figure 4| Expression of ectonucleotidase TMPAP in the sympathoexcitatory region of the ventrolateral medulla prevents sympathetic and cardiovascular responses induced by decreases in cerebral perfusion. Summary data (means ± s.e.m.) illustrating changes in renal sympathetic nerve activity (RSNA), cerebral perfusion pressure (CPP, calculated), heart rate (HR) and mean arterial blood pressure (MAP) induced by increases in intracranial pressure (ICP) in animals transduced to express the control transgene (EGFP) or TMPAP.

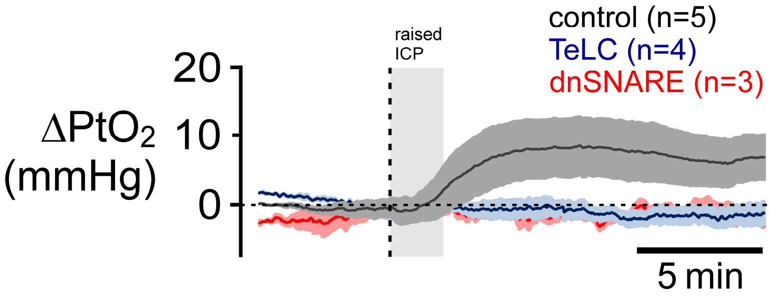

Supplementary Figure 5| Summary data (means ± s.e.m.) illustrating changes in cortical tissue partial pressure of oxygen (PtO_2) induced by increases in intracranial pressure (ICP) in animals transduced to express the control transgene (CatCh-EGFP), TeLC, or dnSNARE in the brainstem astrocytes.

Supplementary Table 1

Hemodynamic variables recorded in groups of rats transduced to express EGFP, dnSNARE, or TeLC in ventral brainstem astrocytes

	N	Circadian phase	**MAP (mmHg)**		1 S.E.M	**SBP (mmHg)**		1 S.E.M	**DBP (mmHg)**		1 S.E.M	**HR (BPM)**		1 S.E.M
EGFP (control)	6	Light phase	101.1	±	2.3	124.7	±	3.2	82.1	±	2.2	368.6	±	11.8
dnSNARE	6	Light phase	100.2	±	1.6	122.8	±	2.3	81.6	±	1.3	369.3	±	7.0
TeLC	3	Light phase	96.5	±	1.9	122.0	±	1.5	77.4	±	2.0	355.9	±	26.9
		Adj. P value (Con. vs. dnSNARE)			0.96			0.88			0.98			1.00
		Adj. P value (Con. vs. TelC)			0.47			0.84			0.37			0.77
EGFP (control)	6	Dark phase	105.7	±	2.9	129.0	±	4.1	87.2	±	2.6	423.7	±	10.6
dnSNARE	6	Dark phase	106.8	±	1.9	129.6	±	2.7	88.2	±	1.4	432.6	±	7.2
TeLC	3	Dark phase	103.1	±	6.7	129.1	±	6.7	83.1	±	6.0	401.8	±	33.0
		Adj. P value (Con. vs. dnSNARE)			0.93			0.99			0.93			0.82
		Adj. P value (Con. vs. TelC)			0.78			1.00			0.46			0.49
