# Supplementary material for: Astrocytes monitor cerebral perfusion and control systemic circulation to maintain brain blood flow
Source: Nat Commun. 2020 Jan 9;11:131. doi: 10.1038/s41467-019-13956-y (PMC6952443; doi:10.1038/s41467-019-13956-y)
Supplement: Supplementary file 3 — Reporting Summary [file 41467_2019_13956_MOESM3_ESM.pdf]

nature research

Corresponding author(s): Gourine

Last updated by author(s): Nov 22, 2019

Reporting Summary

Nature Research wishes to improve the reproducibility of the work that we publish. This form provides structure for consistency and transparency in reporting. For further information on Nature Research policies, see Authors & Referees and the Editorial Policy Checklist.

Statistics

For all statistical analyses, confirm that the following items are present in the figure legend, table legend, main text, or Methods section.

n/a	Confirmed	
☐	☒	The exact sample size (n) for each experimental group/condition, given as a discrete number and unit of measurement
☐	☒	A statement on whether measurements were taken from distinct samples or whether the same sample was measured repeatedly
☐	☒	The statistical test(s) used AND whether they are one- or two-sided<br>*Only common tests should be described solely by name; describe more complex techniques in the Methods section.*
☒	☐	A description of all covariates tested
☒	☐	A description of any assumptions or corrections, such as tests of normality and adjustment for multiple comparisons
☒	☐	A full description of the statistical parameters including central tendency (e.g. means) or other basic estimates (e.g. regression coefficient) AND variation (e.g. standard deviation) or associated estimates of uncertainty (e.g. confidence intervals)
☐	☒	For null hypothesis testing, the test statistic (e.g. F, t, r) with confidence intervals, effect sizes, degrees of freedom and P value noted<br>*Give P values as exact values whenever suitable.*
☒	☐	For Bayesian analysis, information on the choice of priors and Markov chain Monte Carlo settings
☒	☐	For hierarchical and complex designs, identification of the appropriate level for tests and full reporting of outcomes
☒	☐	Estimates of effect sizes (e.g. Cohen's d, Pearson's r), indicating how they were calculated

Our web collection on statistics for biologists contains articles on many of the points above.

Software and code

Policy information about availability of computer code

Data collection	Spike 2.0 version 7; Olympus software; Dataquest A.R.T.; Cellvizio software 2.2
Data analysis	NCSS 2007; µCats Python software (http://doi.org/10.5281/zenodo.1242164; https://github.com/abrazhe/uCats); Dataquest Analysis

For manuscripts utilizing custom algorithms or software that are central to the research but not yet described in published literature, software must be made available to editors/reviewers. We strongly encourage code deposition in a community repository (e.g. GitHub). See the Nature Research guidelines for submitting code & software for further information.

Data

Policy information about availability of data

All manuscripts must include a data availability statement. This statement should provide the following information, where applicable:

- Accession codes, unique identifiers, or web links for publicly available datasets
- A list of figures that have associated raw data
- A description of any restrictions on data availability

Not yet indicated in the text of the manuscript; The data that support the findings of this study are available from the corresponding author upon request.

Field-specific reporting

Please select the one below that is the best fit for your research. If you are not sure, read the appropriate sections before making your selection.

☒ Life sciences ☐ Behavioural & social sciences ☐ Ecological, evolutionary & environmental sciences

For a reference copy of the document with all sections, see nature.com/documents/nr-reporting-summary-flat.pdf

Life sciences study design

All studies must disclose on these points even when the disclosure is negative.

Sample size	The paper describes the results of the experiments performed using in vivo animal preparations. Power calculations for the animal studies are described below. The significance was set at 0.05 and the beta was set at 0.20. From years of relevant research experience, we expect to detect a significant difference with a minimum of 5 animals per group, if the treatments cause difference between means that are as large as 2.25 standard deviations (SD), likely to be a physiologically significant difference. If the difference is as small as 1.75 SD, we increase sample sizes to 9. Although, increasing the sample sizes per group to 15 may enable to detect a difference between means of 1.25 SD, it is doubtful these differences will have a biological significance.
Data exclusions	In the experiment illustrated on Fig 1f, the data obtained in one control animal was excluded from the analysis since the cannula used to deliver the experimental stimulus was blocked. In the experiment illustrated on Fig 4d, the data obtained in 1 control animals, 2 TeLC and 2 dnSNARE animals were excluded from the analysis because either the cannula used to deliver the experimental stimulus was blocked or no transgene expression was detected in the targeted area of the brainstem upon post-hoc histological examination. In the experiment summarized in Supplementary Table 1, the data were not obtained in 3 animals transduced to express TeLC because the batteries in the biotelemetry transducers run out of power during the course of the experiment.
Replication	In our strong opinion, replication of the whole group experiments is not essential for this study. It involved one individual experiment conduced per day and the effect of experimental treatment (ganglionic blockade [Fig 1f], TeLC or dnSNARE expression in the brainstem [Fig 4]) completely abolished the physiological responses in all the individual animals. Assessment of astroglial responses in vivo (Figs 2 and 3) demonstrated robust activations in all the experiments (n=7 and 8, respectively) conduced, therefore we felt that no further tests were required in this study.
Randomization	Randomization is not relevant to this study, since experimental subjects (outbred Sprague-Dawley rats) are obtained from a genetically homogeneous colony and then assigned to different experimental groups according to the treatment.
Blinding	All the experimenters were blinded to the condition or treatment of experimental subjects and to the identity of the experimental groups.

Reporting for specific materials, systems and methods

We require information from authors about some types of materials, experimental systems and methods used in many studies. Here, indicate whether each material, system or method listed is relevant to your study. If you are not sure if a list item applies to your research, read the appropriate section before selecting a response.

Materials & experimental systems

n/a	Involved in the study	
☐	☒	Antibodies
☒	☐	Eukaryotic cell lines
☒	☐	Palaeontology
☐	☒	Animals and other organisms
☒	☐	Human research participants
☒	☐	Clinical data

Methods

n/a	Involved in the study	
☒	☐	ChIP-seq
☒	☐	Flow cytometry
☐	☒	MRI-based neuroimaging

Antibodies

Antibodies used	Chicken anti-GFP (1:500; Aves Labs), rabbit anti-tyrosine hydroxylase (1:500; Santa Cruz), rabbit anti-glial fibrillary acidic protein (1:500; Dako) and mouse anti-microtubule-associated protein 2 (1:500; Sigma) antibodies.
Validation	The antibodies were validated using negative control protocols. We employ these antibodies routinely and we have published numerous research articles demonstrating their specificity. In the present study immunohistochemistry was used to identify anatomical landmarks and the spread of transgene expression, resulting in characteristic staining patterns.

Animals and other organisms

Policy information about studies involving animals; ARRIVE guidelines recommended for reporting animal research

Laboratory animals	Young adult Sprague-Dawley rats (200-300 g)
Wild animals	*Provide details on animals observed in or captured in the field; report species, sex and age where possible. Describe how animals were caught and transported and what happened to captive animals after the study (if killed, explain why and describe method; if released, say where and when) OR state that the study did not involve wild animals.*
Field-collected samples	*For laboratory work with field-collected samples, describe all relevant parameters such as housing, maintenance, temperature, photoperiod and end-of-experiment protocol OR state that the study did not involve samples collected from the field.*

Ethics oversight	All animal experimentations were performed in accordance with the European Commission Directive 86/609/EEC (European Convention for the Protection of Vertebrate Animals used for Experimental and Other Scientific Purposes) and the UK Home Office (Scientific Procedures) Act (1986) with project approval from the University College London Animal Care and Use Committee and the UK Home Office.

Note that full information on the approval of the study protocol must also be provided in the manuscript.

Magnetic resonance imaging

Experimental design

Design type	Changes in cerebral blood flow in response to increased intracranial pressure were recorded in urethane-anesthetized rats
Design specifications	In naïve rats the CBF was measured using ASL MRI. The experimental setup was first pre-calibrated outside of the MRI scanner to deliver increases in ICP by 10-15 mmHg for 30-60 s. FAIR images were acquired every 30 seconds for ~30 minutes with a baseline period of 17 minutes, followed by 30-60 s of raised ICP and 10 minutes of recovery.
Behavioral performance measures	N/A

Acquisition

Imaging type(s)	perfusion
Field strength	9.7 T
Sequence & imaging parameters	A flow sensitive alternating inversion recovery (FAIR) ASL MRI sequence with a three shot segmented EPI readout was applied to measure the cerebralblood flow. The following sequence parameters were used: TR = 5000 ms, TII=2000 ms, matrix size=64×64, FOV=35 mm × 35 mm, TE=10 ms, single slice (slice thickness=2 mm), inversion pulse bandwidth=20 kHz. FAIR images were acquired every 30 s. C
Area of acquisition	Cerebral blood flow time-series data were extracted from manually drawn ROIs in the cortex and the striatum

Diffusion MRI ☐ Used ☒ Not used

Preprocessing

Preprocessing software	*Provide detail on software version and revision number and on specific parameters (model/functions, brain extraction, segmentation, smoothing kernel size, etc.).*
Normalization	*If data were normalized/standardized, describe the approach(es): specify linear or non-linear and define image types used for transformation OR indicate that data were not normalized and explain rationale for lack of normalization.*
Normalization template	*Describe the template used for normalization/transformation, specifying subject space or group standardized space (e.g. original Talairach, MNI305, ICBM152) OR indicate that the data were not normalized.*
Noise and artifact removal	*Describe your procedure(s) for artifact and structured noise removal, specifying motion parameters, tissue signals and physiological signals (heart rate, respiration).*
Volume censoring	*Define your software and/or method and criteria for volume censoring, and state the extent of such censoring.*

Statistical modeling & inference

Model type and settings	*Specify type (mass univariate, multivariate, RSA, predictive, etc.) and describe essential details of the model at the first and second levels (e.g. fixed, random or mixed effects; drift or auto-correlation).*
Effect(s) tested	*Define precise effect in terms of the task or stimulus conditions instead of psychological concepts and indicate whether ANOVA or factorial designs were used.*

Specify type of analysis: ☐ Whole brain ☒ ROI-based ☐ Both

Anatomical location(s): Cortex and Striatum

Statistic type for inference (See Eklund et al. 2016)	*Specify voxel-wise or cluster-wise and report all relevant parameters for cluster-wise methods.*
Correction	*Describe the type of correction and how it is obtained for multiple comparisons (e.g. FWE, FDR, permutation or Monte Carlo).*

Models & analysis

n/a	Involved in the study	
☐	☐	Functional and/or effective connectivity
☐	☐	Graph analysis
☐	☐	Multivariate modeling or predictive analysis

Functional and/or effective connectivity	*Report the measures of dependence used and the model details (e.g. Pearson correlation, partial correlation, mutual information).*
Graph analysis	*Report the dependent variable and connectivity measure, specifying weighted graph or binarized graph, subject- or group-level, and the global and/or node summaries used (e.g. clustering coefficient, efficiency, etc.).*
Multivariate modeling and predictive analysis	*Specify independent variables, features extraction and dimension reduction, model, training and evaluation metrics.*
